# Evaluating the Efficacy of VitalStim Electrical Stimulation Combined with Swallowing Function Training for Treating Dysphagia following an Acute Stroke

**DOI:** 10.6061/clinics/2021/e3069

**Published:** 2021-10-28

**Authors:** Yu Liang, Jing Lin, Hui Wang, Shufen Li, Fang Chen, Lili Chen, Ling Li

**Affiliations:** I Department of Geriatric Rehabilitation, Cadre Sanatorium of Hainan & Geriatric Hospital of Hainan (CSH), Haikou, Hainan 571100, China.; II Eldercare Services and Medical Care Area, Cadre Sanatorium of Hainan & Geriatric Hospital of Hainan (CSH), Haikou, Hainan 571100, China.; III Department of Cardiovascular Medicine, Cadre Sanatorium of Hainan & Geriatric Hospital of Hainan (CSH), Haikou, Hainan 571100, China.; IV Department of Neurology, Cadre Sanatorium of Hainan & Geriatric Hospital of Hainan (CSH), Haikou, Hainan 571100, China.; V Pediatric Area II, Hainan General Hospital, Hainan Affiliated Hospital of Hainan Medical University, Haikou, Hainan 570311, China.

**Keywords:** Electrical Stimulation, Swallowing Function Training, Dysphagia, Stroke, Clinical Efficacy, Quality of Life

## Abstract

**OBJECTIVES:**

This study explored the clinical efficacy of VitalStim electrical stimulation combined with swallowing function training for patients with dysphagia following an acute stroke.

**METHODS:**

Seventy-two patients with dysphagia following an acute stroke were admitted to our hospital and were further divided into two groups using prospective research methods. There were 36 cases in each group according to the random number table method. The control group received conventional medical treatment and swallowing function training while the experimental group received conventional medical treatment and VitalStim electrical stimulation combined with swallowing function training.

**RESULTS:**

The overall response rate of the experimental group (94.44%) was higher than that of the control group (77.78%), and the difference was statistically significant (*p*<0.05). Compared with before treatment, the upward and forward movement speeds of the hyoid bone, anterior movement speed, the grading score of the Kubota drinking water test, Caiteng’s grading score, serum superoxide dismutase, 5-hydroxytryptamine, and norepinephrine levels, Fugl-Meyer Assessment score, and multiple quality of life scores of the two groups showed improvement after treatment. While the standard swallowing assessment score, serum malondialdehyde level, and National Institutes of Health Stroke Scale score decreased, the aforementioned indices showed a significant improvement in the experimental group (*p*<0.05).

**CONCLUSION:**

The results of this study indicate that VitalStim electrical stimulation combined with swallowing function is effective for treating dysphagia following an acute stroke. It can effectively improve swallowing, neurological, and limb motor functions, reduce complications, promote physical recovery, and improve overall quality of life of patients.

## INTRODUCTION

Acute stroke is a common cerebrovascular disease primarily including cerebral infarction and cerebral hemorrhage that can seriously threaten health. Dysphagia is one of the most common complications in patients with acute stroke, with the incidence rate being as high as 47%-50%. The clinical manifestations of patients include a choking cough caused by drinking water and eating difficulties (1). Dysphagia, which can cause malnutrition, aspiration pneumonia, and other conditions, seriously affects the prognosis of patients and lowers their quality of life (2). Currently, swallowing function training is primarily performed for the clinical treatment of dysphagia following an acute stroke, which can be effective in improving the movement of tongue and masticatory muscles and thus improves the swallowing function of patients; however, some patients show poor curative effects (3). Therefore, the effective treatment for dysphagia following an acute stroke remains an important clinical issue. Previously performed empirical studies demonstrated that electrical stimulation for the treatment of dysphagia following an acute stroke can promote throat muscle contraction through depolarization of nerve fibers, excitation of cranial nerves, and restoration of movement control and can therefore improve the swallowing function of patients with fewer adverse reactions and obvious advantages (4,5). As one of the main electrical stimulation therapies used in clinical settings, VitalStim electrical stimulation, which has been extensively implemented in the clinical treatment of dysphagia, is highly effective and portable, and is increasingly recommended by many patients and clinicians (6). In this study, we explore the efficacy of VitalStim electric stimulation combined with swallowing function training for patients with dysphagia following an acute stroke and its effects on indices (e.g., swallowing function and quality of life) so as to provide an important reference for the formulation of clinical protocols for dysphagia following an acute stroke.

## MATERIAL AND METHODS

### General materials

A total of 72 patients with dysphagia following an acute stroke were admitted to our hospital between August 2017 and July 2019 and were divided into two groups using prospective research methods. There were 36 cases in each group according to the random number table method. This study was approved by the Ethics Committee of Hainan General Hospital, Hainan Affiliated Hospital of Hainan Medical University. The inclusion criteria were as follows: first onset, in line with the diagnostic criteria for the acute stroke in *Diagnostic Essentials of Various Cerebrovascular Diseases* (7), clinical symptoms such as eating difficulties and a choking cough caused by drinking water, as confirmed by a head computed tomography or magnetic resonance imaging scan, and swallowing dysfunction of varying degrees identified by two associate chief physicians with rich clinical experience using Kubota drinking water test (KDWT) (8), with the grade of above III; patients aged 18-80 years; and clear mind, stable condition, and voluntary signing of informed consent. The exclusion criteria included: dysphagia because of non-stroke causes; dysarthria and other motor symptoms; combined neck and diseases of pharynx; patients with serious cognitive impairment and consciousness impairment; combined with other cerebral diseases; and poor compliance and cooperation.

### Treatment options

All patients received conventional medical treatment, which primarily included nutrition of brain cells, improvement of microcirculation, anti-platelet aggregation, antihypertensive therapy, and nutritional support, when necessary. The control group received swallowing function training and conventional medical treatment, which included the following: **1)** Cheek muscle training with the following instructions: first, open and close the mouth, take a blow on the chin, then chew on the lower jaw, massage the skin of the affected cheek, and stimulate the cheek with ice cubes. **2)** Tongue muscle training with the following instructions: perform tongue rolling, forward extension, backward contraction, and other movements. If the patient cannot perform autonomous tongue movement, the tongue depressor can be used to gently massage and to perform passive movement. The therapist uses his/her index finger to depress the anterior third part of the patient's tongue, performs horizontal finger tremors for <5s, and helps the patient close his jaw twice/d. **3)** Suction training, which includes putting the index finger of the patient on a rubber sleeve and placing it in the mouth for suction training (15-20 times/time, 5 min/time, two times/d). **4)** Swallowing reflex training where first, the anterior base of the pharyngeal column and base of the upper palate are stimulated with popsicles, and then empty swallowing training is conducted. **5)** Throat training where the head is extended forward, submental muscles are extended for 3-4s, a certain resistance is increased under the chin, the patient is instructed to perform resistance lowering action, the back of tongue is raised against the hard palate, and consonants (e.g., G, K, and ch) are emitted. **6)** Pharyngeal contraction training where the patient is asked to hold their breath after fully inhaling, to swallow saliva slowly, and then to exhale and cough. The swallowing training is performed for four weeks. The experimental group was treated with VitalStim electrical stimulation, swallowing function training, and conventional medical treatment. The instrument used was the VitalStim electrical stimulation therapeutic instrument from the Chattanooga Company, which has a wave width of 700 ms, a frequency of 30-80 Hz, and an intensity of 5-50 mA. During treatment, the two electrodes A and B in channel 1 were horizontally arranged above the hyoid bone, and the two electrodes C and D in channel 2 were horizontally arranged above the upper nail notch and located on both sides of the midline, respectively. The power supply was turned on, and the amplitude of the two channels was slowly increased. The current was adjusted according to the patient’s feedback for 30 min/ time, once /d, five times/week, and this was performed for a total of four weeks.

### Observation indices

The clinical efficacy and swallowing function were evaluated by two associate chief physicians with rich clinical experience to reduce the error in the experiment. To ensure the accuracy of the experimental results, the evaluators were blinded for the group of subjects, and all operations were evaluated in strict accordance with the experimental operating procedures. The clinical efficacy, swallowing function, serum indices [malondialdehyde (MDA), superoxide dismutase (SOD), 5-hydroxytryptamine (5-HT), and norepinephrine (NE)], neurological function, limb motor function, changes in quality of life, and complications were compared between the two groups. **1)** Clinical efficacy. Marked response: the clinical symptoms were notably relieved or disappeared, and the KDWT grade increased by two or more. Response: the clinical symptoms were relieved, and the KDWT grade increased by 1. Non-response: did not meet the above criteria. The overall response rate is the sum of the marked response and response rates. **2)** Swallowing function. The upward and forward movement speeds of the hyoid bone, standard swallowing assessment (SSA) score, grading score of the KDWT, and *Caiteng* grading score were used for evaluation, and the upward and forward movement speeds of the hyoid bone were measured using swallowing radiography; the SSA was performed for pharyngeal reflex, spontaneous cough, laryngeal function, pharyngeal function, and others. Meanwhile, the patient was instructed to swallow 5 ml of purified water, and the observation was performed for laryngeal movement, wheezing during swallowing, and others. If the aforementioned examination results came back normal, the patient was instructed to swallow 60 ml of purified water, and the observation was performed for swallowing time, cough, and others. The total score ranged between 18 and 46 points, and the higher the score, the worse the swallowing function; there were five grades for the KDWT, including Grade I (no choking cough after drinking all the water in one breath, 4 points), Grade II (no choking cough after drinking all the water in two or more breaths, 3 points), Grade III (a choking cough after drinking all the water in one breath, 2 points), Grade IV (a choking cough after drinking all the water in two or more breaths, 1 point), and Grade V (a frequent choking cough and inability to drink water, 0 points). There were seven grades for the Caiteng grading score, including Grade 7 (normal, 6 points), Grade 6 (mild, 5 points), and Grade 5 (stemmatological issues, 4 points), Grade 4 (the chance of swallowing by mistake, 3 points), Grade 3 (swallowing of water by mistake, 2 points), Grade 2 (swallowing of food by mistake, 1 point), and Grade 1 (swallowing saliva by mistake, 0 points). **3)** Serum indices. The fasting venous blood of patients was collected in the morning, the supernatant was taken after centrifugation, the MDA level was determined using colorimetry, the SOD level was detected using the xanthine oxidase method, and the 5-HT and NE levels were determined using enzyme-linked immunosorbent assay. **4)** Neurological and limb motor functions. The evaluation was performed using the National Institutes of Health Stroke Scale (NIHSS) and Fugl-Meyer Assessment (FMA), respectively. It was observed that higher NIHSS scores led to worsening of neurological function and higher FMA scores led to better limb motor function. **5)** Quality of life. The evaluation was performed using the 36-Item Short Form Survey Quality of Life Scale, which included scores on physical pain, physiological function, psychological function, and overall health. Higher scores indicated better quality of life. **6)** Complications. Aspiration pneumonia and a choking cough were included as complications.

### Statistical analyses

SPSS 18.0 software was used for the statistical analysis of the study, and measurement data were expressed using *x̅*±*s*. The comparison between groups was conducted using the independent sample *t* test, and the comparison within groups was conducted using the paired sample *t* test. Numerical data were expressed using percentages, and the *χ*^2^ test was performed with *p*<0.05 indicating statistical significance.

## RESULTS

### Basic data

There was no significant difference between the two groups in terms of sex, mean age, course of disease, lesion site, and stroke (*p*>0.05, Table 1)

### Clinical efficacy

The overall response rate of the experimental group was higher than that of the control group, and the difference was statistically significant (*p*<0.05). This demonstrated that VitalStim electric stimulation combined with swallowing function training is superior to swallowing function training in treating dysphagia following an acute stroke (Table 2).

### The upward and forward movement speeds of the hyoid bone

**Figure 1:**
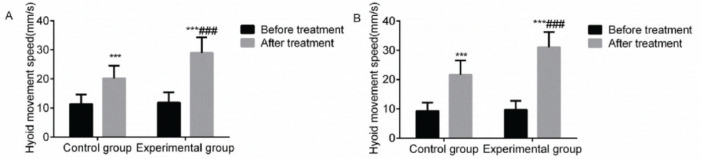
Comparison of the upward and forward movement speeds of the hyoid bone between the two groups before and after treatment. Note: compared with the group before treatment, ***p<0.001; compared with the control group, ^###^ *p*<0.001.

Compared with before treatment, the upward and forward movement speeds of the hyoid bone in the two groups increased after treatment, and a marked rise was observed in the experimental group with a statistically significant difference (*p*<0.05). This indicated that VitalStim electrical stimulation combined with swallowing function training significantly improved the upward and forward movement speeds of the hyoid bone in patients with dysphagia following an acute stroke (Figure 1).

### Swallowing function score

**Figure 2:**
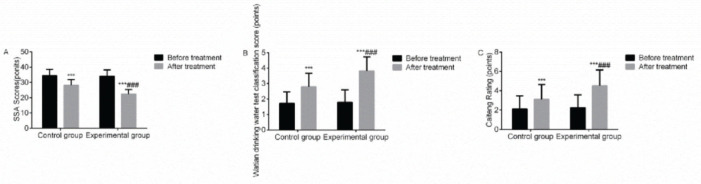
Comparison of the SSA scores, grading scores of the Kubota drinking water test, and Caiteng grading scores between the two groups before and after treatment. Note: compared with the group before treatment, ***p<0.001; compared with the control group, ^###^ *p*<0.001.

Compared with before treatment, the SSA scores of the two groups decreased after treatment, while the Caiteng grading scores and grading scores of the KDWT increased; the increase in the aforementioned indices was more remarkable in the experimental group, with a statistically significant difference (*p*<0.05). It suggested that VitalStim electrical stimulation combined with swallowing function training notably improved the swallowing function of patients with dysphagia following an acute stroke (Figure 2).

### Serum indices

**Figure 3:**
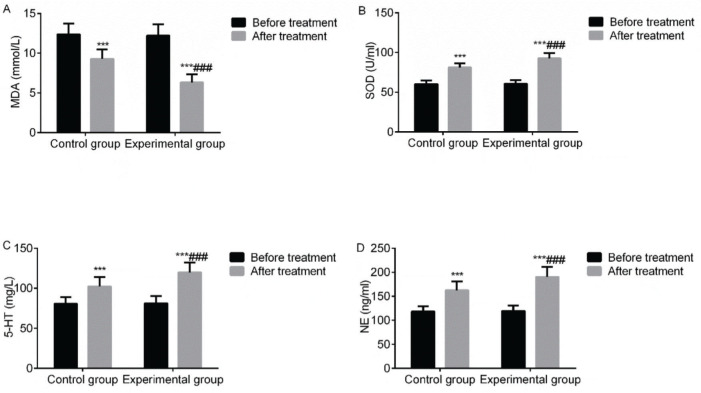
Comparison of the changes in serum indices between the two groups before and after treatment. Note: compared with the group before treatment, ***p<0.001; compared with the control group, ^###^ *p*<0.001.

Compared with before treatment, the levels of serum MDA in the two groups decreased after treatment, while the levels of serum SOD, 5-HT, and NE increased. The aforementioned indices in the experimental group improved significantly (*p*<0.05). It demonstrated that VitalStim electrical stimulation combined with swallowing function training significantly improved the levels of serum MDA, SOD, 5-HT and NE of patients with dysphagia following an acute stroke (Figure 3).

### Neurological and limb motor functions

**Figure 4:**
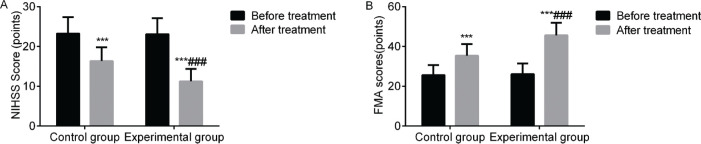
Comparison of the NIHSS and FMA scores between the two groups before and after treatment. Note: compared with the group before treatment, ***p<0.001; compared with the control group, ^###^ *p*<0.001.

Compared with before treatment, the NIHSS scores of the two groups decreased after treatment, while the FMA scores increased. The aforementioned indices in the experimental group improved significantly (*p*<0.05). This indicated that VitalStim electrical stimulation combined with swallowing function training considerably improved the neurological and limb functions of patients with dysphagia following an acute stroke (Figure 4).

### Complications

The incidence rate of complications in the experimental group was lower than that in the control group, and the difference was statistically significant (*p*<0.05). The results indicated that VitalStim electric stimulation combined with swallowing function training was superior to swallowing function training in treating dysphagia following an acute stroke, with fewer complications (Table 3).

### Quality of life

**Figure 5:**
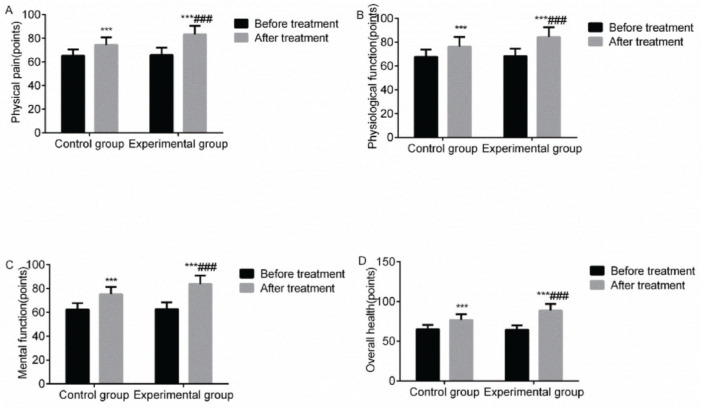
Comparison of the quality of life between the two groups before and after treatment. Note: compared with the group before treatment, ***p<0.001; compared with the control group, ^###^ *p*<0.001.

Compared with before treatment, the quality of life scores of the two groups increased after treatment, and there was a marked increase in the experimental group, with a statistically significant difference (*p*<0.05). This highlighted that VitalStim electrical stimulation combined with swallowing function training can significantly improve the quality of life in patients with dysphagia following an acute stroke (Figure 5).

## DISCUSSION

Over the past few years, the aging population in China has been on the rise, and as such, there has been an increase in the number of patients with stroke as well. A stroke features a sudden onset of symptoms and a relatively short time for rescue, and it can often be accompanied by complications of varying degrees which, thus, takes its toll on the patient’s families and society at large (9). As one of the most common complications of acute stroke, dysphagia is also known as functional dysphagia and is predominantly caused by the degeneration of reflective activities of cerebrovascular nerves, resulting in uncoordinated swallowing muscles, aspiration, a choking cough, and other conditions. Additionally, it increases the risk of aspiration pneumonia and malnutrition in patients. In severe cases, it can lead to airway obstruction and even asphyxia which, ultimately, increases the mortality rate of patients (10,11). Therefore, effective improvement of the swallowing function of patients with dysphagia following an acute stroke can actively reduce the occurrence of aspiration pneumonia and malnutrition and lead to an improvement in the prognosis and quality of life of patients (12,13). Clinically, oxidative stress can aggravate cerebral tissue damage in patients with dysphagia following an acute stroke, and MDA and SOD can reflect the degree of oxidative stress in patients (14). It has been previously reported that a stroke can indirectly or directly destroy the metabolism, synthesis, and conduction pathways of 5-HT and NE, resulting in a decrease in serum 5-HT and NE levels, further hindering information conduction and affecting swallowing function (15). Therefore, improvement in the serum levels of MDA, SOD, 5-HT and NE in patients with dysphagia following an acute stroke is of great significance to relieve the patient's condition, to alleviate cerebral tissue damage, and to promote the recovery of swallowing function. Reports have shown that electrical stimulation may regulate the energy mechanism of patients with stroke, promote the balance of neurotransmitters and neuropeptides, improve the central function of swallowing cortex, enhance blood oxygen activity, and thus improve the swallowing function of patients (16-18).

In this study, VitalStim electrical stimulation combined with swallowing function training was used to treat patients with dysphagia following an acute stroke. The results suggested that the overall response rate of the experimental group was higher than that of the control group, while the incidence rate of complications in the experimental group was lower than that of the control group. Additionally, the upward and forward movement speeds of the hyoid bone, the grading scores of the KDWT, Caiteng grading score, serum SOD, 5-HT, and NE levels, FMA score, and multiple quality of life scores of the two groups all increased after treatment; on the other hand, the SSA score, serum MDA level, and NIHSS score all decreased, and the aforementioned indices in the experimental group significantly improved. The findings are, at the most part, consistent with the results reported previously (19). It is thought that VitalStim electrical stimulation combined with swallowing function training for the treatment of patients with dysphagia following an acute stroke could significantly improve clinical efficacy, swallowing function, neurological and limb motor functions, serum SOD, 5-HT, and NE levels, FMA score level, and quality of life while also reducing the occurrence of complications in patients. This should be attributed to the reason that swallowing function training for patients with dysphagia after acute stroke could be targeted to train multiple muscles, increase coordination and flexibility of muscles, prevent the disuse muscular atrophy of pharyngeal muscles, reflexively stimulate the central nervous system, promote nerve cell regeneration and functional reorganization, and thus improve swallowing function of patients (20-22). As a neuromuscular electrical stimulation technique, VitalStim electrical stimulation, which has been commonly used to treat patients with dysphagia following an acute stroke, can activate synapses that are dormant or inhibited, effectively stimulate the central nervous system, promote the recovery of the swallowing reflex control function, reconstruct a reflex arc, prevent pharyngeal muscles from disuse and muscular atrophy at an earlier stage, induce pharyngeal muscles to contract autonomously, promote the recovery of motor function in articulation muscles and swallowing muscles, and increase muscle strength, thus improving swallowing function of patients (5,23). Additionally, VitalStim electrical stimulation is a safe and effective treatment method. During the treatment, no abnormal changes were observed in the patients’ pulse, blood pressure, and others and there were no laryngeal spasms (24).

In summary, VitalStim electrical stimulation combined with swallowing function training, which is effective in the treatment of patients with dysphagia following an acute stroke, can effectively improve swallowing function, neurological and limb motor functions, quality of life, reduce complications, and promote physical recovery of patients. However, because of the small sample set and short observation time in this study, the results may be biased. Future multi-center studies with a larger number of cases and longer observation time are needed to shed further light on this topic.

## AUTHOR CONTRIBUTIONS

Liang Y, Lin J and Li L conceived and designed the research, and interpreted the results of experiments. Liang Y, Lin J, Wang H, Li S, Chen F, Chen L, Li L performed the experiments, analyzed the data, prepared the figures, and drafted the manuscript. All authors approved final version of manuscript.

## Figures and Tables

**Table 1:** Comparison of basic data between the two groups.

Group	Sex (%)		Mean age (*x̅*±*s*, years)	Mean course of disease (*x̅*±*s*, d)	Lesion site (%)		Stroke type (%)		Mean NIHSS score, point	Combined diseases		Mean BMI (kg/m^2^)
	Male	Female			Brainstem	Hemisphere	Cerebral hemorrhage	Cerebral infarction		Hypertension	Diabetes	
Control group (n=36)	20 (55.56)	16 (44.44)	63.14±5.49	31.85±6.34	13 (36.11)	23 (63.89)	15 (41.67)	21 (58.33)	23.25±4.12	24 (66.67)	18 (50.00)	24.38±2.35
Experimental group (n=36)	22 (61.11)	14 (38.89)	62.74±6.14	31.27±5.85	12 (33.33)	24 (66.67)	13 (36.11)	23 (63.89)	23.10±4.04	23 (63.89)	16 (44.44)	24.57±2.42
*t*	0.229		0.291	0.403	0.061		0.234		0.156	0.028		0.338
*p*	0.633		0.772	0.688	0.805		0.629		0.877	0.867		0.736

**Table 2:** Comparison of clinical efficacy between the two groups [n (%)].

Group	Marked response	Response	Non-response	Overall response rate
Control group (n=36)	15 (41.67)	13 (36.11)	8 (22.22)	28 (77.78)
Experimental group (n=36)	20 (55.56)	14 (66.67)	2 (5.56)	34 (94.44)
*χ* ^2^	-	-	-	4.181
*p*	-	-	-	0.041

**Table 3:** Comparison of incidence rate of complications between the two groups [n (%)].

Group	Aspiration pneumonia	Cough	Total
Control group (n=36)	4 (11.11)	5 (13.89)	9 (25.00)
Experimental group (n=36)	0 (0.00)	1 (2.78)	1 (2.78)
*χ*^2^	-	-	7.432
*p*	-	-	0.006
